# Supplementary material for: Mapping the evolution of fertility support policies in China: A content and instrumental analysis
Source: PLoS One. 2025 Oct 9;20(10):e0332137. doi: 10.1371/journal.pone.0332137 (PMC12510515; doi:10.1371/journal.pone.0332137)
Supplement: S1 Appendix — (ZIP) [file pone.0332137.s001.zip › S1 Appendix. 226 original policy documents/209-第十届全国人民代表大会第五次会议关于2006年中央和地方预算执行情况与2007年中央和地方预算的决议(FBM-CLI-1-89365).doc]

第十届全国人民代表大会第五次会议关于2006年中央和地方预算执行情况与2007年中央和地方预算的决议

**发布部门：** [全国人民代表大会](https://www.pkulaw.com/law/chl?Aggs.IssueDepartment=101)

**发布日期：**2007.03.16

**实施日期：**2007.03.16

**时效性：** [现行有效](https://www.pkulaw.com/law/chl?Aggs.TimelinessDic=01)

**效力级别：** [工作文件](https://www.pkulaw.com/law/chl?Aggs.EffectivenessDic=XA0105)

**法规类别：** [机关工作综合规定](https://www.pkulaw.com/law/chl?Aggs.Category=00301) [预算管理](https://www.pkulaw.com/law/chl?Aggs.Category=03126)

第十届全国人民代表大会第五次会议关于2006年中央和地方预算执行情况与2007年中央和地方预算的决议
（2007年3月16日第十届全国人民代表大会第五次会议通过）

　　第十届全国人民代表大会第五次会议审查了国务院提出的《关于2006年中央和地方预算执行情况与2007年中央和地方预算草案的报告》及2007年中央和地方预算草案，同意全国人民代表大会财政经济委员会的审查结果报告。会议决定，批准《关于2006年中央和地方预算执行情况与2007年中央和地方预算草案的报告》，批准2007年中央预算。

关于2006年中央和地方预算执行情况与2007年中央和地方预算草案的报告
--2007年3月5日在第十届全国人民代表大会第五次会议上
财　政　部

各位代表：
　　受国务院委托，现将2006年中央和地方预算执行情况与2007年中央和地方预算草案提请十届全国人大五次会议审议，并请全国政协各位委员提出意见。
　　**一、**2006年中央和地方预算执行情况
　　2006年，在党中央、国务院的坚强领导下，各地区、各部门以科学发展观为统领，认真贯彻中央各项方针政策和十届全国人大四次会议的有关决定、决议，实现了“十一五”时期的良好开局，经济建设和各项社会事业发展取得了新的巨大成就，人民生活进一步改善，中央和地方预算执行情况也比较好。
　　全国财政收入39343.62亿元（不含债务收入，下同），比2005年增加7694.33亿元，增长24.3%，比预算超收3920.24亿元，其中，中央财政本级收入超收2542.47亿元，地方财政本级收入超收1377.77亿元。按规定解决出口退税历史陈欠613亿元后（账务上以退库处理），全国财政收入为38730.62亿元，比2005年增加7081.33亿元，增长22.4%，比预算超收3307.24亿元，完成预算的109.3%。全国财政支出40213.16亿元，比2005年增加6282.88亿元，增长18.5%，完成预算的104.8%。
　　分中央与地方来看：中央财政总收入21232.31亿元（已扣除解决出口退税历史陈欠613亿元），比2005年增加3971.82亿元，增长23%，比预算超收1960.27亿元，完成预算的110.2%。其中，中央本级收入20449.77亿元，比2005年增加3901.24亿元，增长23.6%，比预算超收1929.47亿元，完成预算的110.4%；地方上解收入782.54亿元，比2005年增加70.58亿元，增长9.9%，比预算超收30.8亿元，完成预算的104.1%。中央财政总支出23482.26亿元，比2005年增加3222.27亿元，增长15.9%，完成预算的105.7%。其中，中央本级支出9991.56亿元，比2005年增加1215.59亿元，增长13.9%，完成预算的104.9%；对地方税收返还和转移支付13490.7亿元，比2005年增加2006.68亿元，增长17.5%，完成预算的106.3%。新建立中央预算稳定调节基金500亿元。中央财政赤字2749.95亿元，比十届全国人大四次会议批准的2950亿元减少200.05亿元，占GDP的比重下降为1.3%。2006年年末中央财政国债余额控制在35381.68亿元的限额以内。中央政府性基金收入1706.87亿元，支出1706.87亿元。地方财政总收入31771.55亿元，比2005年增加5186.77亿元，增长19.5%，完成预算的107.3%。其中，地方本级收入18280.85亿元，比2005年增加3180.09亿元，增长21.1%，完成预算的108.2%。地方财政总支出31004.14亿元，比2005年增加5137.87亿元，增长19.9%，完成预算的104.7%。其中，地方本级支出30221.6亿元，比2005年增加5067.29亿元，增长20.1%，完成预算的104.8%；上解中央支出782.54亿元，比2005年增加70.58亿元，增长9.9%，完成预算的104.1%。地方财政收支相抵，结余或结转767.41亿元。上述预算执行数字在决算编制汇总后，还会有些小的变化。
　　2006年中央预算执行主要情况是：
　　（一）依法加强收入征管，财政收入保持较快增长。2006年，国民经济保持了增长速度较快、效益较好、物价水平较低的态势。在此基础上，各级财政、税务、海关等部门依法加强收入征管，积极实施科学管理，全面清理和规范税收优惠政策，严格控制减免税，努力做到应收尽收，中央和地方财政收入都保持了较快增长。财政收入形势好主要是经济形势好的反映，也表明财政收入机制渐趋完善，财政发展具有一定的稳定性和协调性。需要说明的是，除经济形势好、效益提高这一关键因素外，财政收入较快增长也存在一次性、政策性和超常规因素。主要包括：中国银行、中国建设银行等国有商业银行股改后利润增加，所得税大幅增收；开征石油特别收益金；探矿权采矿权价款收入集中入库；受固定资产投资和工业生产增长较快、资源性产品价格上扬等影响，相关行业税收超常规增长。剔除这些因素，财政收入增长和经济发展情况是基本相适应的。
　　按照党中央、国务院的统一部署和安排，根据《[预算法](https://www.pkulaw.com/chl/b2d319164ed40178bdfb.html)》、《[全国人大常委会关于加强中央预算审查监督的决定](https://www.pkulaw.com/chl/e191a6c4c04c5a51bdfb.html)》及十届全国人大四次会议有关决议的要求，中央财政超收收入2573亿元（未扣除解决出口退税历史陈欠），除按有关法律、法规和财政体制规定，增加对地方税收返还和转移支付231亿元、增加教育支出16亿元外，主要用于加强经济社会发展的薄弱环节、解决历史欠账和削减财政赤字，以增强财政经济发展的可持续性。具体是：增加社会保障支出241亿元；增加石油特别收益金专项支出372亿元，用于对农渔林等行业因油价上涨增支的补助，以及能源开发、节能、环保等方面的重大项目支出；增加预算内经常性投资100亿元，主要用于支持社会主义新农村建设；增加专项支出135亿元，用于公路建设、购置储备油和大中型水库移民后期扶持；增加“三奖一补”资金、对西藏农牧民补助、缉私经费补助、口岸专项转移支付35亿元；增加出口退税指标613亿元，用于解决剩余出口退税历史陈欠；减少中央财政赤字200亿元；预留2007年调整工资及相关支出130亿元。为更加科学合理地编制预算，保持中央预算的稳定性和财政政策的连续性，安排500亿元新建立中央预算稳定调节基金。中央预算稳定调节基金的安排使用纳入预算管理，接受全国人大及其常委会的监督。2006年中央财政预计超收收入安排使用的情况，国务院已向全国人大常委会报告。
　　（二）综合运用多种财税手段，促进经济平稳较快发展。2006年，根据经济形势的发展变化，在实施稳健财政政策过程中，进一步调整和丰富了政策手段，并有促有控，增强财政调控的有效性和针对性。一是积极调整和完善相关税收政策。从2006年4月1日起，对消费税的税目和税率进行了1994年以来最大规模的有增有减的调整。将个人所得税工薪所得费用减除标准和内资企业所得税计税工资税前扣除标准由800元/人月提高至1600元/人月。调整了资源税政策。调整部分产品的出口退税率，扩大了“高耗能、高污染”产品取消出口退税和降低退税率的范围，同时增补加工贸易禁止类商品目录，对部分“高耗能、高污染”产品加征出口关税，降低部分资源性产品进口关税。东北地区增值税转型改革试点等税制改革工作也稳步推进。税收政策的调整和完善，对促进资源节约和环境保护，合理引导消费和间接调节收入分配都发挥了积极作用。二是相机调整中央政府投资规模并优化使用结构。2006年中央政府投资总规模1254亿元（含超收安排100亿元），主要用于社会主义新农村建设、社会事业、环境生态、西部大开发等方面的项目，适当开工建设关系发展全局的重大项目。同时根据宏观经济运行状况合理把握预算下达进度，严格投资管理，加强监督，提高了投资使用效益。三是调整规范住房土地收支管理的财税政策。将2005年实施的个人购房转手交易征免营业税期限由2年延长至5年。完善住房公积金管理制度，进一步明确和落实城镇廉租住房保障的资金来源。有针对性地调整新增建设用地土地有偿使用费征收标准和使用管理政策，制定规范国有土地使用权出让收支管理的相关政策，初步遏制了房地产市场中的投机行为，进一步贯彻落实了最严格的耕地保护制度，促进了节约集约用地。四是研究实施有利于节能和开发新能源的财税政策。针对石油价格高企的新形势，积极研究促进发展石油替代能源的措施，制定支持生物能源发展等扶持政策，发展替代能源的财税政策体系初步建立。同时积极推进石油价格形成机制改革，开征石油特别收益金。这些措施与其他调控措施相配合，有力地推动了经济向宏观调控的预期方向发展。
　　（三）加大财政支农力度，社会主义新农村建设开局良好。2006年财政支持“三农”具有力度大、政策实、措施多、机制新的特点，公共财政的阳光越来越多地照耀到农村。2006年中央财政用于“三农”的各项支出共3397亿元（不包括用石油特别收益金安排的对种粮农民综合直补120亿元），比2005年增加422亿元，增长14.2%。一是全面取消农业税。同时，取消了农业特产税，制定出台《[烟叶税暂行条例](https://www.pkulaw.com/chl/d2fb69ebe899ddc9bdfb.html)》，这标志着国家与农民的传统分配关系发生了根本性变革，有利于农民减负增收和休养生息，有利于农业的长远发展。中央和地方财政分别安排转移支付782亿元和250亿元。与改革前的1999年相比，农民减负约1250亿元，人均减负约140元，农场农工负担也进一步减轻。农村税费改革进入农村综合改革的新阶段。二是加大补贴政策实施力度。中央财政新增120亿元补贴资金，适时对种粮农民因农业生产资料价格上涨导致的增支实行综合直补，7.28亿种粮农民直接受益，并根据石油价格调整情况，拨付渔业等行业补贴资金85.9亿元。全国粮食直补资金达到142亿元，比2005年增加10亿元，其中13个粮食主产省（区）126.8亿元，均占本省（区）粮食风险基金的50%以上。中央财政安排良种补贴41.5亿元、农机具购置补贴6亿元，其中，农机具购置补贴资金比2005年增长100%。完善粮食最低收购价政策，及时拨付补贴资金59亿元。全面推广“一卡（折）通”，将补贴资金直接发放到农民的银行卡或存折中，并启动中国农民补贴网建设，加强补贴管理。符合我国国情、综合补贴与专项补贴相结合、管理比较规范的农业补贴政策体系初步建立，保障和促进了粮食安全生产。三是顺利实施农村义务教育经费保障机制改革。从2006年春季学期开始西部地区率先进行改革，全部免除农村义务教育阶段学生学杂费，同时对家庭经济困难学生免费提供教科书，并补助寄宿生生活费；中央财政对西部地区农村义务教育阶段中小学安排公用经费补助，启动全国农村义务教育阶段中小学校舍维修改造长效机制。中部地区每个省选择一个县于秋季学期开始试点，东部地区大部分省份也主动实施改革。中央和地方财政分别安排资金150亿元和211亿元，并对部分专项资金实行国库集中支付，资金直达学校，平均每学年每个小学生减负140元、初中生减负180元。这一改革逐步将农村义务教育纳入公共财政保障范围，说明农村公共产品供给方式正在发生深刻变革。在此基础上，大力支持实施“农村义务教育阶段学校教师特设岗位计划”，并在全国十个省份启动“新农村卫生新校园建设工程”试点，为加快农村义务教育发展注入了新的活力。四是扩大新型农村合作医疗制度改革试点范围。据统计，全国50.7%的县（市、区）进行了改革试点，参合农民4.1亿人，中央财政补助标准由10元提高到20元，省级财政也相应提高补助标准。中央财政安排补助资金42.7亿元。从制度和机制上缓解了农民群众“因病致贫、因病返贫”的问题。五是大力支持农业生产和农村长远发展。重点支持农村“六小”工程等项目建设，着力改善农村生产生活条件。深入推进农业综合开发，改造中低产田、扶持产业化经营等，预计新增粮食生产能力29.1亿公斤；中央财政研究确定从2006年起，用4年时间核减农业综合开发债务44.86亿元，进一步调动农民发展生产的积极性。中央财政安排扶贫资金137亿元，重点支持贫困乡村基础设施建设、农村贫困劳动力转移培训和农业产业化发展。大力支持农业技术推广、农民专业合作组织发展、农村劳动力转移培训、启动新型农民科技培训工程和“科普惠农兴村计划”、推进“科技富民强县专项行动计划”等。六是积极支持建立多元化的支农投入机制。在全国162个县开展整合支农资金试点，提高财政支农资金使用的规模效益。积极探索建立“民办公助”等机制，调动农民投入的积极性。大力支持农村金融体系建设。2006年，中央财政拨付试点地区农村信用社保值贴补利息资金29.31亿元，并将试点地区的所得税减免政策延长3年，帮助农村信用社逐步化解历史包袱，增强发展后劲，更好发挥社会主义新农村建设生力军的作用。这些措施促进了农业生产、农民增收和农村发展，加快了城乡协调发展步伐。
　　（四）重点支出得到保障，各项社会事业加快发展。切实体现公共财政公共性和公益性的本质特征，财政支出重点向社会事业倾斜，突出加强社会发展的薄弱环节，促进经济社会协调发展。一是积极支持就业再就业工作和社会保障体系建设。2006年全国财政就业和社会保障支出4337.65亿元，增长17.3%，完成预算的109.1%。其中，中央财政社会保障支出2010.02亿元，增长22.9%（详见《中华人民共和国2006年全国预算执行情况2007年全国预算（草案）》，以下简称预算草案，第52页）。在东北三省试点的基础上，增加8个省份开展做实企业职工基本养老保险个人账户试点。推动城市最低生活保障制度与就业再就业政策的合理衔接。大力支持中西部地区农村医疗救助制度建设、困难地区城市医疗救助试点和农村五保供养工作。认真落实积极的就业政策，完善实施办法，支持国有困难企业妥善处理历史遗留问题。积极配合研究解决企业军转干部和军队退役人员的安置问题。特别是面对自然灾害比较严重的形势，中央财政安排并及时拨付各种抗灾救灾资金112亿元，地方财政也积极安排和拨付抗灾救灾资金，有力地支持了抗灾救灾。二是支持教育事业发展。2006年全国财政教育支出4752.7亿元，增长19.6%，完成预算的103.8%。其中，中央财政教育支出536亿元，增长39.4%（详见预算草案第51页）。在重点支持农村义务教育的同时，启动实施国家示范性高等职业院校建设计划，支持职业院校实训基地建设。继续支持实施“985工程”等，提高高等教育质量。完善以国家助学贷款、助学金、奖学金等为主要资助手段的高校和中等职业教育家庭经济困难学生资助政策体系，帮助家庭经济困难学生顺利完成学业。三是支持卫生事业发展。2006年，全国财政医疗卫生支出1311.58亿元，增长26.5%，完成预算的110.7%。其中，中央财政医疗卫生支出138亿元，增长65.4%（详见预算草案第53页）。重点加大公共卫生专项资金的投入力度，支持艾滋病、结核病、血吸虫病等重大传染病和地方病防治、贫困孕产妇住院分娩、基层卫生机构设备配备和人员培训、万名医师支援农村卫生工程等。四是支持科技创新。2006年，建立财政科技投入的稳定增长机制，全国财政科技支出1260.38亿元，增长26.2%，完成预算的110.2%。其中，中央财政科技支出774亿元，增长29.2%（详见预算草案第50页）。进一步优化投入结构，增加对基础研究、应用研究的投入，加大了对公益性科研机构的支持力度。出台激励性的财务制度，制定税收优惠、政府采购等财税政策措施，支持企业自主创新。五是支持文化事业和产业发展。2006年，全国财政文体广播事业费支出834.53亿元，增长18.6%，完成预算的108.5%。其中，中央财政文体广播事业费支出123亿元，增长23.9%。主要用于积极推进文化体制改革，进一步完善公共文化服务体系，重点实施广播电视村村通工程、农村电影数字化放映、全国文化信息共享、农村部分计划生育家庭奖励扶助制度、西部地区计划生育“少生快富”等工程。六是支持生态建设和环境保护。完善天然林保护工程政策，积极推进退耕还林后续政策调整等，完善森林生态效益补偿基金制度，支持国有林场管理体制和国有林权制度改革试点。全年安排退耕还林400万亩、退牧还草1.5亿亩。大力推进煤炭资源有偿使用制度改革，从2006年起8个省份已全面推开改革试点。这些措施进一步表明我国社会事业正处在一个加速发展的新时期。
　　（五）着力缓解县乡财政困难，促进基本公共服务均等化。认真落实促进区域协调发展的各项财税优惠政策，特别是继续加大转移支付力度，缓解基层财政困难。一是加大“三奖一补”政策实施力度。2006年奖补资金规模达到235亿元，比2005年增加85亿元，并完善奖补机制。巩固和扩大了缓解县乡财政困难成果，全国财政困难县的总数已经由2005年初的791个大幅度减少到目前的200个左右，并将持续向好的方向发展。推动了省以下财政管理方式创新，全国28个省份推行乡财县管改革，18个省份推行省直管县改革，为缓解县乡财政困难提供了体制和机制保障。进一步调动了产粮大县生产粮食的积极性，维护了粮食安全。提高了县乡政府的行政能力，维护了社会稳定。二是完善一般性转移支付稳定增长的长效机制。2006年中央财政用于对地方主要是中西部地区的一般性转移支付资金达到1527亿元，比2005年增加407亿元。三是完善民族地区转移支付办法。2006年民族地区转移支付达到155.63亿元，并将非民族自治区、州的民族自治县纳入民族地区转移支付范围。完善了革命老区、边疆地区、西藏等困难地区转移支付办法。四是支持加强基层政权建设。2006年中央财政安排政法补助专款59.3亿元，帮助地方基层政法部门进一步改善执法办案条件。安排监狱体制改革经费9.25亿元，推动监狱体制改革。特别是为适应建设社会主义新农村的需要，从2006年开始，用两年时间支持解决全国98086个村级组织无活动场所的问题，中央财政2006年已安排4.9亿元。此外，首次实现了大中型水库移民后期扶持政策的统一，大力支持库区和移民安置区经济发展。这些措施提高了地方特别是基层政府提供基本公共服务的能力。
　　（六）大力支持各项改革，社会主义市场经济体制不断完善。预算管理制度改革及支持一些重点领域和关键环节的改革又取得新的重要进展。一是改革公务员工资制度、规范公务员收入分配秩序进展顺利。实施了公务员、军人、驻外使领馆工作人员、驻港澳地区内派工作人员工资制度改革，相应调整提高了机关事业单位离退休人员、企业离休人员和援外出国人员工资生活待遇，提高了企业退休人员、优抚对象和城市低保对象等的待遇水平和生活补助标准，并对具有高级职称的企业退休人员，部分退休早、基本养老金水平相对偏低的人员予以倾斜，这是历史上提高幅度以及中央财政补助规模最大的一次。同时，全面清理整顿了公务员津贴补贴，建立并逐步完善了监督制约机制。二是大力支持企业改革和发展。中央财政安排补助资金250亿元，支持93户国有企业实施政策性破产，妥善安置职工41万人。核定经费补助基数48.1亿元，初步完成第二批74户中央管理企业分离办社会工作任务。东北地区厂办大集体改革试点工作也顺利起步。同时，出台一系列财税政策，支持中小企业发展，推动外贸改革和实施“走出去”战略。三是积极推动金融体制改革。摸清了金融类国有资产底数和分布情况。制定了国有股权益上缴政策、国有股减持政策和所得税政策，支持中国银行、中国工商银行顺利改制上市。四是稳步推进政府收支分类改革。这是建国以来我国财政收支分类统计体系最为重大的一次调整，也是我国政府预算管理制度的又一次深刻创新。新的政府收支分类体系充分体现了国际通行做法与国内实际的有机结合，有利于更加清晰完整地反映政府收支全貌和职能活动情况，对进一步提高政府预算透明度，强化预算管理与监督，从源头上治理腐败，促进社会主义民主政治建设等，都具有十分重要的意义。五是不断深化其他预算管理制度改革。结余资金管理加强，绩效考评试点工作稳步推进，定员定额试点不断完善，项目预算实现滚动管理。国库集中支付改革范围扩大到所有中央部门及所属6100多个基层预算单位，实施改革的预算资金达到4600多亿元。36个省、自治区、直辖市、计划单列市本级，270多个地（市），1000多个县（区），超过16万个基层预算单位也实施了此项改革。非税收入收缴管理制度改革进展加快，改革范围不断扩大。全国政府采购规模达到3500亿元，在节能、环保等方面较好发挥了扶持和导向作用。政府采购领域商业贿赂专项治理工作取得阶段性成果。中央国库现金管理稳妥开展，国债余额管理顺利实施，金财工程建设步伐加快。这些措施有力地推动了传统预算管理制度向现代预算管理制度转变，促进了社会主义市场经济体制的不断完善。
　　（七）严格依法理财，财政管理进一步规范。扎实贯彻依法治国基本方略，把依法理财贯穿财政工作的始终。一是财政法律制度体系不断完善。《[企业所得税法（草案）](https://www.pkulaw.com/chl/07764981f678c159bdfb.html)》将在本次大会上进行审议，《[烟叶税暂行条例](https://www.pkulaw.com/chl/d2fb69ebe899ddc9bdfb.html)》、《[车船税暂行条例](https://www.pkulaw.com/chl/b7ecd4a9bdafbb6abdfb.html)》、《[城镇土地使用税暂行条例](https://www.pkulaw.com/chl/0ac5b0fcf44ad8d4bdfb.html)》已由国务院发布或修订发布。《[行政单位国有资产管理暂行办法](https://www.pkulaw.com/chl/96046940ac47f706bdfb.html)》和《[事业单位国有资产管理暂行办法](https://www.pkulaw.com/chl/f1a78311fe535d08bdfb.html)》正式发布，行政事业单位国有资产管理开始步入法制化、规范化的轨道。建立了科学规范的彩票公益金分配制度。财政行政审批制度改革不断深化，中央财政行政许可事项各项制度及申请等均在网上公示。二是会计等基础建设继续强化。建立了与经济发展进程相适应、与国际标准趋同的会计、审计两大准则体系。同时实施会计领军人才培养工程，完善继续教育制度。研究提出了我国政府会计改革战略框架总体设想。注册会计师和资产评估行业监管加强，业务领域进一步扩大。三是财政监督成效明显。初步建立了实时监控、综合核查、整改反馈、跟踪问效的财政监督机制，重点监督涉及人民群众切身利益的重大支出使用情况。共查处有问题资金911亿元，挽回损失150亿元。四是整改工作不断加强。国务院对于全国人大有关决议和审计报告所提意见和问题高度重视，要求自觉接受审计监督，认真落实审计决定，坚决纠正存在的问题。目前，各部门和有关单位已认真进行了整改。审计发现的中央预算执行中存在的问题，大部分已得到整改纠正并追究有关人员的责任。有关整改情况国务院已报全国人大常委会。
　　2006年中央预算执行和财政经济运行中也存在一些亟待解决的问题。一是经济结构性矛盾比较突出，对“三农”、教育、卫生等经济社会发展薄弱环节的支持力度仍需加大。二是对解决涉及国计民生问题的投入力度仍需继续加大，如：就业形势依然严峻，农村低保制度尚未完全建立，部分地区还存在“上学难、上学贵”以及“看病难、看病贵”的问题，等等。三是经济发展的代价较大，对提高自主创新能力、推进转变经济增长方式的扶持政策措施仍需加强。四是部分地区基层财政还比较困难，基层公共服务的财政保障能力需继续提高。五是财政资金损失浪费等现象时有发生，财政收支管理的规范化、科学化和现代化水平仍有待提升。我们将增强紧迫感、使命感，着力深化改革，优化支出结构，完善相关制度，严肃财经纪律，加强监督检查，不断提高财政管理和服务水平。

　　**二、**2007年中央和地方预算草案
　　根据中央对2007年工作的总体部署，结合面临的新形势，2007年预算编制和财政工作的总体思路是：以邓小平理论和“三个代表”重要思想为指导，认真贯彻党的十六大、十六届三中、四中、五中、六中全会和中央经济工作会议精神，全面落实科学发展观，加快构建社会主义和谐社会，加强调控，完善措施，继续实施稳健财政政策，着力促进经济又好又快发展；以人为本，突出重点，调整优化财政支出结构，着力支持社会发展和解决民生问题；加大力度，健全制度，规范省以下财政管理体制，着力促进地区间基本公共服务均等化；勇于探索，敢于创新，积极深化各项改革，着力完善财税体制机制，更好地让全体人民享受改革发展成果和公共财政阳光。
　　根据上述总体思路，2007年预算的主要指标安排如下：中央财政总收入24421.08亿元，比2006年增加3188.77亿元，增长15%。其中，中央本级收入23590.3亿元，比2006年增加3140.53亿元，增长15.4%，这个增幅高于税务、海关部门的税收计划420亿元，执行中如果完不成预算收入，则通过调入中央预算稳定调节基金解决；地方上解收入830.78亿元，比2006年增加48.24亿元，增长6.2%。中央财政总支出26871.08亿元，比2006年增加3388.82亿元，增长14.4%。其中，中央本级支出11062亿元，比2006年增加1070.44亿元，增长10.7%；对地方税收返还和转移支付15809.08亿元，比2006年增加2318.38亿元，增长17.2%。中央财政收支相抵，赤字2450亿元。中央财政国债余额限额37865.53亿元，比2006年增加2483.85亿元。汇总中央和地方财政收支安排，2007年全国财政收入44064.85亿元，比2006年增加5334.23亿元，增长13.8%。全国财政支出46514.85亿元，比2006年增加6301.69亿元，增长15.7%。财政收入按高于经济增长速度安排，既考虑了我国经济社会较快发展的趋势和近几年收入增长的实际，也考虑了各种减收因素，是积极稳妥的。
　　需要说明的是，提交大会审议的2007年预算草案及指标是按照新的政府收支分类科目编制的，为便于代表审议，我们另行在2007年预算草案表中对2006年预算执行数按新科目作了相应调整。
　　2007年中央财政重点支出安排和主要财税政策如下：
　　（一）继续适当减少中央财政赤字，调整和优化政府投资使用方向与结构。宏观调控是经济保持平稳较快发展的客观需要。根据中央经济工作会议精神和经济发展的新形势，2007年中央财政赤字调整为2450亿元，比2006年预算赤字减少500亿元，预计占GDP的比重继续下降到1.1%。中央政府投资总规模安排1304亿元，比2006年预算的1154亿元增加150亿元。其中，国债项目资金规模调整为500亿元，比2006年减少100亿元；预算内基本建设投资规模调整为804亿元，比2006年预算增加250亿元。进一步调整和优化国债项目资金及中央预算内投资的使用方向与结构，向经济社会发展薄弱环节倾斜，重点用于改善农村公共服务。
　　（二）强化各项支农惠农财税政策，加快社会主义新农村建设。建设社会主义新农村是构建社会主义和谐社会的重要内容，也是促进城乡协调发展和经济发展的重大举措。扎实贯彻“多予少取放活”的方针，坚持把支持解决“三农”问题作为预算安排和财政工作的重中之重。2007年中央财政用于“三农”的各项支出共达3917亿元，比2006年增加520亿元，增长15.3%。一是严格执行增加投入政策。新增政府投资主要用于社会主义新农村建设，确保用于直接改善农村生产生活条件的投入比2006年有所增加。继续落实新增教育、卫生、文化支出主要用于农村的政策，提高车购税支出用于农村的比重。土地出让收入在确保足额支付征地和拆迁补偿费后，重点向社会主义新农村建设倾斜，逐步提高用于农业土地开发和农村基础设施建设的比重。二是加大支农补贴力度。中央财政安排良种补贴55.7亿元、农机具购置补贴12亿元，分别比2006年增加14.2亿元、6亿元，并将科学改进补贴政策和方式。进一步完善粮食直补、农资综合直补办法，探索建立粮食综合补贴制度。落实完善粮食最低收购价政策，稳定农民种粮收益。三是支持在全国范围建立农村最低生活保障制度。中央财政安排农村最低生活保障补助支出30亿元，用于鼓励已经建立制度的地区完善制度，支持未建立制度的地区积极探索建立制度。各地区根据当地经济发展水平和财力可能，确定低保对象范围、标准，中央财政对财政困难地区给予适当补助。四是促进现代农业建设。把发展生产放在更加突出的位置。中央财政安排经费5亿元，用于农业技术推广。安排试点补助9亿元，支持测土配方施肥。安排补助资金3亿元，促进农业产业化及农产品加工业发展。安排农业综合开发支出115亿元，并引导地方财政和社会资金投入，着力提高农业综合生产能力和农业综合效益。安排补助资金10亿元，完善“民办公助”机制，支持小型农田水利设施建设。支持畜牧业健康发展，推进农业结构调整。安排经费11亿元，支持农村劳动力转移就业培训和新型农民科技技能培训，提高农业生产经营者素质。五是扩大农村综合改革试点范围。鼓励有条件的地方在全省范围内开展农村综合改革试点，暂不具备条件的省份也要扩大市、县改革试点范围。认真清理化解乡村债务，扩大化解乡村债务试点范围，建立激励约束机制，优先化解举办农村义务教育等公益事业形成的债务。全面落实国有农场税费改革政策。六是创新财政扶贫开发机制。安排扶贫支出144亿元，比2006年增加7亿元。总结完善贫困村村级发展互助资金试点工作经验，积极探索建立以结果为导向的扶贫资金分配激励机制、贫困县进退机制，提高扶贫开发成效。七是支持农村文化事业发展。中央财政安排文化体育与传媒支出173亿元，比2006年增加47.13亿元，增长37.4%。安排资金25亿元，加快实施广播电视村村通工程，切实提高中央广播电视节目无线覆盖水平，支持解决农民群众“听广播难、看电视难”的问题。以农村基层为重点，积极推进全国文化信息资源共享工程和农村电影数字化放映。八是促进深化农村金融体制改革。中央财政安排试点地区农村信用社保值贴补利息资金19.56亿元，支持深化农村信用社改革。安排农业保险保费补贴资金10亿元，选择部分农业保险基础较好的农业大省，支持开展政策性农业保险试点。同时，加大支农资金整合力度，注重发挥政府资金的带动作用，引导农民和社会资金投入农村建设，加快构建多元化的社会主义新农村建设投入稳定增长机制。
　　（三）大力推进机制和政策创新，着力支持解决“上学难、上学贵”的问题。教育影响人生走向，涉及千家万户，惠及子孙后代，关系国家未来。2007年，中央财政安排教育支出858.54亿元，比2006年增加252.49亿元，增长41.7%（详见预算草案第51页）。一是在全国范围内推行农村义务教育经费保障机制改革。在巩固西部地区改革成果的基础上，2007年中部和东部地区也全面推行，对全国1.5亿农村义务教育阶段中小学生全部落实免除学杂费政策，并提高农村义务教育阶段中小学公用经费保障水平，同时，对家庭经济困难学生免费提供教科书并补助寄宿生生活费。中央财政相应安排资金279.8亿元。继续支持开展“农村义务教育阶段学校教师特设岗位计划”、“新农村卫生新校园建设工程”试点，深入研究建立城市义务教育经费保障机制实施方案。二是全面建立完善家庭经济困难学生资助政策体系。建立健全普通本科高校、高等职业学校和中等职业学校国家奖学金、助学金制度，并在教育部直属师范大学实行师范生免费教育。2007年，中央财政安排资金95.1亿元，用于扩大政策受益面，提高资助标准。争取在“十一五”期间建立起系统、科学、公平、有效的家庭经济困难学生资助政策体系，从制度上基本解决家庭经济困难学生“上不起学”的问题，促进教育公平。同时，突出政策导向，资助重点向中西部地区倾斜，向农村地区倾斜，向职业教育倾斜，向国家最需要的专业倾斜，促进教育结构的调整和优化，培养社会需要的多元化合格劳动者，引导和促进就业，避免“千军万马走独木桥”。上述政策和机制的全面实施，标志着我国已从义务教育这个基础环节和经济困难学生这个贫困群体入手，着手建立比较完善的财政保障政策体系框架，将更加有效地解决“上学难、上学贵”这个突出问题。中央财政还将大力支持职业教育发展和提高高等教育质量。
　　（四）多种措施和政策并举，积极支持解决“看病难、看病贵”的问题。健康关系人民群众幸福生活，是人全面发展和经济社会可持续发展的基础。2007年，中央财政安排医疗卫生支出312.76亿元，比2006年增加145.36亿元，增长86.8%（详见预算草案第53页）。一是实施免费防治重大传染病政策。努力做到能预防的预防，能治疗的治疗，能监控的切实加强监控。将可以通过接种疫苗有效预防的法定重大传染病纳入国家免疫规划范围，并在原来免费救治艾滋病、结核病、血吸虫病患者的基础上，将救治效果好的麻风病等纳入免费救治范围。加大重大传染病防控经费保障力度，支持开展传染病防控知识培训，提高传染病防控水平。改变过去以地方财政为主负担法定重大传染病防治资金的机制，实行以中央财政负担为主的新机制。这一政策不仅可以使更多患者直接受益，而且将更好地控制疾病的传播，使全社会受益。二是加快新型农村合作医疗制度建设步伐。2007年，新型农村合作医疗制度试点范围扩大到全国80%以上的县（市、区），提前一年基本在全国范围内建立起新型农村合作医疗制度。中央财政对中西部地区继续按每人20元的标准给予补助，对东部地区给予适当补助，地方财政补助标准也要全部提高到每人20元，并不断完善办法，规范管理，确保参合农民真正受益，并且得到越来越多的实惠。三是支持开展城镇居民基本医疗保险试点。从2007年开始进行城镇居民基本医疗保险试点，逐步将城镇职工之外的城镇居民纳入基本医疗保险覆盖范围，中央财政将对中西部地区的困难城镇居民参保缴费给予适当补助。同时，扩大城镇职工基本医疗保险的覆盖范围，支持做好关闭破产和困难国有企业退休人员参保工作，使广大人民群众有钱看病。四是加大城乡医疗救助力度。2007年，中央财政将继续加大投入，支持中西部地区完善农村医疗救助制度，稳步推进城市医疗救助试点，推动城乡医疗救助工作的规范化和制度化，逐步提高救助水平，加强医疗救助基金使用管理，切实减轻城乡困难群众看病就医的经济负担。五是大力支持提高基层卫生服务能力。进一步加大对农村卫生和城市社区卫生人员培养的支持力度，力争到2010年将农村卫生和城市社区卫生人员全部轮训一遍，所需资金主要由中央财政和地方财政负担。同时，建立城市社区公共卫生经费保障机制，中央财政从2007年起对中、西部地区城市社区服务人口分别按人均3元、4元的标准给予补助，使城市居民安全、有效、方便、快捷地享受健康教育、预防、保健、康复、计划生育技术服务等基本公共卫生服务。六是大力支持中医药振兴发展。加大支持“名医、名科、名院”建设和适宜中医药技术在基层推广应用的力度，促进中医药振兴发展，更好地发挥中医药的特色和优势。同时，大力支持加强食品药品监管能力建设。
　　（五）突出帮助解决基本民生问题，切实加强就业再就业和社会保障等工作。大力支持解决民生问题，是促进社会公平、构建社会主义和谐社会最基本的要求。公共财政作为公共资源配置的主体，其最大、最基本的作用就是要体现社会的公平和正义。一是加大社会保障和就业再就业投入力度。2007年，中央财政安排社会保障和就业支出2019.27亿元，在去年较大幅度增加的基础上又比2006年增加246.99亿元，增长13.9%（详见预算草案第52页）。二是继续推动其他社保工作。完善城市居民最低生活保障和企业职工基本养老保险制度，支持扩大做实基本养老保险个人账户试点工作。继续落实促进就业再就业的各项财税扶持政策，加快解决历史遗留问题。以建立健全社会保险基金的财务制度和监督制约机制为重点，进一步加强对社保基金以及其他各类社会保障资金的管理。支持做好农民工及被征地农民就业和社会保障工作。完善社会救助体系，大力支持抗灾救灾，积极帮助解决困难群众的生产生活问题。三是继续规范收入分配秩序。严格执行公务员工资制度改革、规范公务员收入分配秩序有关政策规定。根据国务院确定的企业退休人员养老金调整方案，继续提高企业退休人员基本养老金水平。确保中央改革规范政策落实到位，切实防止出现新的拖欠。完善企业收入分配政策，调节国有企业管理者与职工的收入差距，加强对垄断行业收入分配的监管，规范垄断企业收入分配制度。四是支持城镇廉租住房保障制度建设。落实预算安排、住房公积金增值收益以及土地出让收益等各项城镇廉租住房保障资金来源，通过租金补贴、租金核减和实物配租等方式，着力支持解决低收入家庭住房困难问题，使困难群体居有其所。
　　（六）完善财税政策，加快经济增长方式转变。提高自主创新能力，促进资源节约和环境友好是转变经济增长方式的重要环节和突破口，也是实现经济可持续发展的必由之路。一是加大科技投入力度。2007年，中央财政安排科学技术支出881.21亿元，比2006年增长20.1%（详见预算草案第50页）。优化中央财政科技投入结构，继续加大对公益性科研机构的支持力度，推动科研机构深化管理体制改革。支持重大科技专项的实施。推动建设各具特色和优势的区域创新体系。二是增强企业自主创新能力。充分利用税收扶持政策，整合资金，鼓励和引导企业增加研究开发投入。促进知识产权保护。鼓励创业风险投资对中小高新技术企业进行投资。支持关键核心技术及重大技术装备的自主研发和国产化。推进建立产学研有机结合的新机制。拓展政府采购政策对自主创新的扶持功能等。三是稳步推进资源、环境有偿使用制度改革试点。完善煤炭资源有偿使用制度改革试点，选择电力行业和太湖流域开展排污权有偿使用和排污权交易试点，研究建立跨省流域生态补偿机制。加大对生态建设和环境保护的投入，研究出台鼓励废旧物资回收，发展可再生能源、替代能源和新能源等的相关财税政策，完善矿产资源补偿费和排污收费政策，加快建立比较完善的鼓励能源、资源节约和环境保护的财税政策体系。四是完善出口退税和关税政策。巩固出口退税机制改革成果，研究实施鼓励资源性和有利于技术创新商品的进口，以及控制“高耗能、高污染”商品出口的税收政策。支持企业提高运用国际贸易规则的能力。
　　（七）积极配合企业所得税两法合并等改革，进一步完善社会主义市场经济体制。更有效地发挥财税制度调节国民经济运行、促进经济结构调整和可持续发展的作用。一是积极配合做好内外资企业所得税两法合并立法工作。研究制订[企业所得税法](https://www.pkulaw.com/chl/07764981f678c159bdfb.html)实施条例及相关配套政策措施，保障《[企业所得税法](https://www.pkulaw.com/chl/07764981f678c159bdfb.html)》顺利实施，促进公平竞争和企业做大做强，扩大内需，推动经济的可持续发展。二是稳步推进其他税制改革。抓紧研究制定全面实施增值税转型改革的具体方案及配套措施。出台实施新的资源税制度。力争完成并实施新的耕地占用税。研究开征物业税的实施方案。抓紧完善燃油税改革方案并择机实施。研究修订印花税条例。不断完善房地产涉税调控相关政策措施。三是大力支持深化国有企业、金融体制等改革。继续支持做好国有企业政策性关闭破产，妥善安置关闭破产企业人员，推动经济结构调整。加快推进东北地区厂办大集体改革试点。支持中小企业发展。积极推进政策性银行、中国农业银行、出口信用保险公司的改革，推进金融资产管理公司商业化转型。四是开展国有资本经营预算制度试点。编制国有资本经营预算，有利于增强政府的宏观调控能力，减除国有企业发展中的体制性障碍，推动国有经济布局和结构的战略性调整，支付必要的改革成本，并规范国家与国有企业的分配关系。中央财政将选择中央直管企业和烟草企业，开展编制试点，地方财政也要积极开展试点，并确保试点取得预期成效。
　　（八）加大转移支付力度，提高地方政府提供基本公共服务的能力。继续贯彻落实西部大开发、振兴东北地区等老工业基地、促进中部地区崛起的财税政策，支持东部地区率先发展，并不断加大财政支持力度，不仅有利于提高政府公共服务能力，也有利于加快经济结构的优化调整。一是加大一般性转移支付力度。2007年中央财政对地方主要是中西部地区的一般性转移支付资金将达到1924亿元，对民族地区转移支付资金将达到210亿元，比2006年分别增加397亿元和54亿元。二是发展和扩大缓解县乡财政困难成果。2007年中央财政安排奖补资金335亿元，比2006年增加100亿元。完善“三奖一补”政策，同时对各地工作情况进行检查考评，确保政策落到实处、资金使用合理。抓紧完善省以下财政管理体制，积极推进省直管县和乡财县管等改革。三是认真落实司法经费保障政策。中央财政安排贫困地区公检法补助64.9亿元，比2006年增加5.6亿元，并完善管理制度，建立激励机制。抓紧研究解决诉讼费办法改革后的法院经费保障问题。地方财政也要落实好司法经费保障政策，尽快将司法机关履行职能所需经费列入本级预算予以全额保证。2007年，中央财政安排公共安全支出594.83亿元，比2006年增加131.25亿元，增长28.3%。
　　另外，为提高我军在高技术条件下的防卫作战和应对突发事件的能力，维护国家主权和领土完整，适当提高军人待遇，安排国防支出3472.32亿元，比2006年增加524.98亿元，增长17.8%；为加强国际间交流与合作，安排外交支出230.66亿元，比2006年增加62.71亿元，增长37.3%；为加强中央政权建设和支付国债利息，安排一般公共服务支出2160.53亿元，比2006年增加67.95亿元，增长3.2%。中央财政还将积极保障服务改革发展稳定大局的其他重点支出需要。

　　**三、**扎实工作，确保圆满完成2007年预算
　　根据2007年政府工作的总体要求，为确保圆满完成2007年预算，在统筹兼顾的基础上，将重点抓好以下工作：
　　（一）狠抓税收、非税收入征管，促进财政收入稳定增长。在大力支持经济发展、做大经济“蛋糕”的基础上，依法加强收入征管。大力整顿和规范税收秩序，严厉打击各种偷骗税等违法活动，严格控制减免税，抓紧清理到期的税收优惠政策，坚决制止和纠正擅自出台“先征后返”等变相减免税政策。在全国稳步推进财税库行税收收入电子缴库横向联网，进一步强化增值税、企业所得税、个人所得税等各税种的征收管理。稳步推进税式支出制度改革试点。全面清理和规范非税收入，坚决取消不合理和不合法的非税收入项目，完善非税收入管理政策和制度，重点研究制定《政府性基金管理办法》、《政府非税收入征收管理办法》等。将土地出让收支全部纳入地方政府性基金预算，强化土地出让收支监督管理，进一步加强土地、海域、矿产等资源性收入的征收管理。加强行政事业单位国有资产收益管理，将行政单位国有资产有偿使用收入和处置收入，事业单位国有资产处置收入等纳入财政非税收入管理，实行“收支两条线”，抓好并不断推进开展个人收入与单位的行政权力和管理的国有资产彻底脱钩的试点工作，从源头上促进规范收入分配秩序。努力做到应收尽收，确保财政收入稳定增长。
　　（二）狠抓预算管理制度改革，进一步提高财政管理的科学化、现代化水平。及时总结政府收支分类改革经验，根据出现的新情况、新问题，完善科目设置，研究新科目体系下的预算管理模式。在建立健全支出标准体系等的基础上，在农业部等14个中央部门（企业）开展项目支出按经济分类编制预算试点，进一步细化预算编制。同时，深入研究改进政府预算报表，使政府预算真正做到反映全面、公开透明、便于监督。继续加强结余资金管理，积极推进绩效考评试点，建立健全支出标准体系，将参照[公务员法](https://www.pkulaw.com/chl/823ca97b7f55d18cbdfb.html)管理的事业单位纳入定员定额试点范围，实现实物费用定额与定员定额之间的有效衔接，研究建立资产管理与预算管理有机结合的机制。中央和省级国库集中支付改革要扩大到所有基层预算单位，中央级政府性基金、离退休经费和省级政府性基金要纳入改革范围；所有地市力争都实行这一改革，县级也要积极推进。中央部门本级及执收单位都要实施非税收入收缴改革，省级改革的资金范围要扩大到所有非税收入，省以下也要积极推进。进一步健全政府采购制度体系，扩大政府采购规模，加大推进专项拨款政府采购实施力度，切实发挥政府采购政策功能，规范政府采购行为。加强行政事业单位国有资产管理。开展行政事业单位资产清查，出台行政事业单位国有资产管理的规章制度，初步建成全国行政事业单位资产动态监管信息系统。加快推进金财工程建设，把先进的信息技术手段更好地融入到财政管理中，提高预算编制和管理的水平。
　　（三）狠抓重点支出保障，切实规范专项资金管理。中央财政和地方各级财政都要严格按照科学发展观和构建社会主义和谐社会的要求，大力调整支出结构，上下共同努力，切实保障党中央、国务院确定的各项重点支出需要，扎扎实实地体现在预算安排和预算执行中。抓紧推进专项资金的规范管理，争取尽快取得明显进展。严格控制新专款项目的设立，除国家法律、法规及国务院文件有明确规定的以外，中央财政新设立专款项目要经国务院批准。规范专项转移支付分配，完善资金管理办法，努力做到公开、透明、规范。清理整合专项转移支付项目，尽可能减少项目支出的交叉、重复，真正体现专项转移支付的特定目标。改进专项转移支付预算编制，逐步将专项转移支付列入各级地方预算，同时，推进专项转移支付资金国库集中支付试点。
　　（四）狠抓依法理财，加强财政监督管理。全面推进法治财政建设，深入贯彻国务院《[全面推进依法行政实施纲要](https://www.pkulaw.com/chl/61052ca84cd0d03fbdfb.html)》，积极做好[企业所得税法](https://www.pkulaw.com/chl/07764981f678c159bdfb.html)实施条例、[政府采购法](https://www.pkulaw.com/chl/6b1222b4fc405df6bdfb.html)实施条例、评估行业管理条例、企业国有资本经营预算管理条例、彩票管理条例等财税立法工作。认真抓好会计准则、审计准则、财务通则的实施，加强和完善会计、审计、资产评估等基础工作，大力推进企业内控标准体系建设。加快政府会计改革研究步伐。继续实施会计人才培养工程，加强注册和监管，促进注册会计师和资产评估行业健康发展。健全财政监督机制，加大对重大财政政策执行过程的监督力度，着力监督涉及群众切身利益的财政资金使用情况。自觉接受全国人大和审计、纪检部门的监督以及社会团体和舆论的监督。牢固树立依法理财和为民理财的意识，预防各类违反财经法规问题的发生。
　　（五）狠抓勤俭节约，坚决反对铺张浪费。尽管近年来经济总量越来越大、财政收入快速增长，但仍需保持清醒头脑，居安思危，增强忧患意识。我国还是世界上最大的发展中国家，仍然并将长期处于社会主义初级阶段，发展很不平衡，生活困难群众数量较多，人民群众的生活还不富裕，国家建设需要办的事情很多，绝不能因为财政收入形势相对好一些就可以大手大脚花钱。要切实按照建立节约型社会和“两个务必”的要求，严格按预算控制支出特别是一般性支出的增长，改革完善公务活动接待制度，严格差旅费和会议费管理，对中央国家机关出差、办会实行定点管理办法。严肃财经纪律，加大监管力度，厉行节约，勤俭办一切事业，坚决反对铺张浪费，真正把钱用在刀刃上，切实提高财政资金使用的规范性、安全性和有效性。
　　2007年是深入贯彻落实科学发展观、积极推进社会主义和谐社会建设的重要一年。圆满完成预算和各项财政工作任务意义重大。我们要在以胡锦涛同志为总书记的党中央领导下，高举邓小平理论和“三个代表”重要思想伟大旗帜，认真贯彻落实科学发展观，按照十届全国人大五次会议对经济财政工作提出的各项要求，自觉接受人民代表大会对财政工作的监督和指导，认真听取人民政协的意见和建议，坚定信心，齐心协力，奋发进取，扎实工作，为全面建设小康社会和加快构建社会主义和谐社会做出更大贡献，以新的成绩迎接党的十七大胜利召开！

第十届全国人民代表大会财政经济委员会关于
2006年中央和地方预算执行情况与2007年中央和地方预算草案的审查结果报告
(2007年3月12日第十届全国人民代表大会第五次会议主席团第二次会议通过)

十届全国人大五次会议主席团：
　　第十届全国人民代表大会第五次会议审查了国务院提出的《关于2006年中央和地方预算执行情况与2007年中央和地方预算草案的报告》以及2007年中央和地方预算草案。全国人民代表大会财政经济委员会在对预算报告和预算草案进行初步审查的基础上，根据各代表团和有关专门委员会的审查意见又作了进一步审查。国务院根据审查意见对预算报告作了修改。现将审查结果报告如下。

　　**一、**财政经济委员会认为，2006年预算执行情况是好的。2006年，全国财政收入39343亿元（不含债务收入），扣除解决出口退税历史陈欠613亿元后为38730亿元，超过预算3307亿元，完成预算的109.3%；全国财政支出40213亿元，超过预算1840亿元，完成预算的104.8%。中央财政总收入21232亿元（已扣除解决出口退税历史陈欠613亿元），超过预算1960亿元，完成预算的110.2%；中央财政总支出23482亿元，超过预算1260亿元，完成预算的105.7%。新建立中央预算稳定调节基金500亿元。中央财政赤字2749.95亿元，比预算减少200.05亿元。2006年末，中央财政国债余额为35015亿元，在十届全国人大四次会议批准的35568亿元限额之内。地方财政收支相抵，结余或结转767亿元。
　　2006年12月，国务院向全国人大常委会作了中央财政预计超收收入安排使用情况的报告。中央财政超收收入主要用于增加对地方税收返还和转移支付，增加社保、教育、农业方面支出，冲减赤字和解决出口退税历史陈欠，建立中央预算稳定调节基金等。
　　2006年，国务院和地方各级人民政府全面落实党中央的方针政策和十届全国人大四次会议提出的各项要求，认真落实科学发展观，继续实施稳健的财政政策，财政收入增长较快，财政对重点支出的保障力度进一步加大，国家财政在构建社会主义和谐社会中发挥了重要作用。同时应当看到，预算执行和财政运行中还存在一些必须认真解决的问题。主要是：预算执行结果与预算差异过大，预算编制的准确性和科学性不高，固定资产投资、科技等预算资金年初分配到项目的到位率不高，预算执行中追加较多，影响了预算的严肃性和约束性；财政对“三农”、卫生、社会保障等经济社会事业的支持力度还需加强；财政转移支付制度不尽完善，专项转移支付的比重仍然较高；部分地区县乡财政还比较困难；行政成本偏高；铺张浪费现象仍然比较严重等，财政管理监督有待进一步加强。

　　**二、**2007年的预算安排，全国财政收入44064亿元，比上年增加5334亿元，增长13.8%；全国财政支出46514亿元，比上年增加6301亿元，增长15.7%。中央财政总收入24421亿元，比上年增加3188亿元，增长15.0%；中央财政总支出26871亿元，比上年增加3388亿元，增长14.4%。中央财政收支相抵，赤字2450亿元。2007年末中央财政国债余额限额37865亿元。
　　财政经济委员会认为，2007年中央预算草案，体现了落实科学发展观、促进经济又好又快发展和构建社会主义和谐社会的要求。继续实施稳健的财政政策，调整优化支出结构，着力支持社会事业发展和解决民生问题。预算草案是可行的。
　　财政经济委员会建议，批准《2006年中央和地方预算执行情况与2007年中央和地方预算草案的报告》，批准2007年中央预算草案。地方各级政府预算由本级人民代表大会审查批准。

　　**三、**为更好地完成2007年预算，做好财政工作，财政经济委员会提出以下建议：
　　（一）加快建立财政支农资金稳定增长的长效机制
　　我国农业和农村发展仍处在比较困难的阶段。中央财政和地方财政都要切实做到支农资金增量高于上年、政府固定资产投资用于农村的增量高于上年、土地出让收入用于农村建设的增量高于上年，做到财政支持“三农”的支出占当年财政支出的比重不降低。要加快建立各级政府财政支农分工负责的保障机制，增强财政支农的稳定性和有效性。研究建立中央和地方财政支农的统一的统计制度。政府部门要转变职能，加大整合支农资金力度，扩大整合范围，提高整合层次，解决支农资金多头分散、重复安排、效益不高的问题。要积极发挥财政支农资金的带动作用，引导社会资金对“三农”的投入。要利用财政贴息、补助等手段，为开展金融支农和发展农业保险创造有利条件。
　　（二）加大解决民生问题的力度
　　要抓住当前经济发展较快和财政增收较多的有利时机，坚持以人为本，继续加大财政投入，积极解决就业、社会保障、教育、卫生医疗、住房等民生问题。切实做好今年全面实施农村义务教育经费保障机制改革工作，建立经济困难学生的资助政策体系，优先化解农村“普九”形成的历史债务。要将基本养老、基本医疗、失业、工伤和生育等社会保险基金纳入预算管理，研究编制社会保障预算。研究改革社会保险费征管体制，加强保险基金监督管理，确保资金安全。在做好城市居民最低生活保障工作的同时，要积极支持在全国范围建立农村最低生活保障制度，支持做好农民工及被征地农民就业和社会保障工作。要强化政府责任，提高财政资金对公共卫生的投入，逐步建立覆盖城乡居民的基本卫生保健制度。加快新型农村合作医疗制度和以社区为基础的新型城市卫生服务体系建设，加快开展城镇居民基本医疗保险试点和推广，做好关闭破产和困难国有企业退休人员参加基本医疗保险工作。
　　（三）完善节约能源资源和保护环境的财税政策
　　各级财政要加大对节能减排的支持力度，要将节约能源资源、保护环境作为财税政策支持的重点。改革资源税制度，逐步将水资源纳入资源税征税范围，对原油、天然气和煤炭资源税的计税依据由从量计征改为从价计征。要制定和完善鼓励节能产品和治污技术的研究、开发、生产和使用的财税政策。大力支持发展新能源和可再生能源。研究对废气、污水、固体废物等征收环境税的问题。
　　（四）规范财政转移支付制度
　　在加大转移支付力度的同时，调整转移支付结构，降低专项转移支付比重，提高财力性转移支付比重，提高地方政府公共服务能力和保障水平，促进基本公共服务的均等化。地方各级要将上级的税收返还和转移支付编入本级预算。进一步完善财政转移支付制度，逐步实现规范化，切实做到公开、透明。
　　（五）深化财税体制改革
　　要提高预算编制的科学性和准确性，改变预算执行结果与预算安排差异过大的问题。要切实解决固定资产投资、科技等预算资金年初到位率低的问题，提前做好项目的储备，没有准备好的项目，不要列入当年预算。要做好政府收支分类改革工作，并及时总结完善。细化预算编制，提交全国人大审查的预算草案要编列到款。进一步深化部门预算改革，强化部门预算的约束性。做好编制国有资本经营预算的试点工作，规范国家与国有企业的收入分配关系。加快增值税由生产型转为消费型的改革。认真总结分税制改革以来的经验，研究建立健全与事权相匹配的财税体制。加强税收法制建设，将成熟的税收条例上升为法律。
　　（六）进一步加强财政管理和审计监督
　　要严肃财经纪律，加强财政财务管理，提高财政资金使用效益。要发扬艰苦奋斗、厉行节约的精神，严格控制行政性支出，反对铺张浪费。反对搞各种“形象工程”、“政绩工程”。要加强税收征管，做到应收尽收。要加强对非税收入的预算管理，制定非税收入纳入预算的目录，改变目前非税收入纳入预算的随意性。要加强对中央预算和部门预算执行的审计，特别要加强对项目支出、财政专项转移支付、预算超收收入、非税收入、预算追加和收支两条线执行等的审计。今年应对社会保险基金、土地出让收入、金融资产管理公司等关系群众切身利益、对经济和社会影响大的项目，开展审计调查和监督，推进政府及部门依法行政，依法理财。
　　以上报告，请予审议。

全国人民代表大会财政经济委员会
　　2007年3月12日

© 北大法宝：（[www.pkulaw.com](https://www.pkulaw.com/)) 专业提供法律信息、法学知识和法律软件领域各类解决方案。北大法宝为您提供丰富的参考资料，正式引用法规条文时请与标准文本核对。
欢迎查看所有[产品和服务](https://www.pkulaw.com/helps/2.html)。法宝快讯：[如何快速找到您需要的检索结果？](https://www.pkulaw.com/helps/70.html)[法宝](https://www.pkulaw.com/helps/69.html)V6有何新特色？

扫描二维码阅读原文

**原文链接：**<https://www.pkulaw.com/chl/4fcdd47fe380a7f5bdfb.html>
